# First Reported Case of 'Epidermal Nevus Syndrome' with a Triad of Central Nervous System Deformities

**DOI:** 10.7759/cureus.916

**Published:** 2016-12-06

**Authors:** Waqas Ullah, Hafez Mohammad A Abdullah, Muhammad A Shahzad, Muhammad Aslam Sadiq, Ejaz Ahmad, Sana Khan

**Affiliations:** 1 Internal Medicine, University of Arizona, Tucson, AZ; 2 Internal Medicine, Khyber Teaching Hospital, Peshawar, Pakistan; 3 Internal Medicine, Hayatabad Medical Complex, Peshawar, Pakistan; 4 Internal Medicine, Nishtar Hospital, Multan, Pakistan; 5 Internal Medicine, Griffin Hospital

**Keywords:** epidermal nevus syndrome, cortical dysplasia, dandy walker, cerebellar atrophy

## Abstract

Epidermal nevus syndrome (ENS) is a term used to describe the occurrence of an epidermal nevus in association with other extra-cutaneous developmental anomalies, most commonly involving the nervous and musculoskeletal systems. The nevus is classified on the basis of the main component which may be keratinocytic, sebaceous, follicular, apocrine, or eccrine. Most patients who present with ENS is at the time of birth, though some become apparent later in life. This case describes a young female who presented with seizures and cognitive impairment along with a linear epidermal nevus on the midline of her face. The presence of the nevus prompted brain imaging which showed cortical dysplasia, multiple hamartomas in the temporal lobe, thalamus, and periventricular regions along with cerebellar atrophy and Dandy-Walker variant. To our knowledge, this is the first case in which three different types of brain lesions were found in the same patient.

## Introduction

The association of epidermal nevi with neurologic symptoms was first established by Gustav Schimmelpenning [1]. The initially described constellation of findings included sebaceous nevi in association with neurological findings like seizures, mental retardation, and ocular coloboma. However, several other findings such as ocular hemangiomas and optic gliomas have also been described. Hemimegalencephaly is the most common brain malformation reported in conjunction with epidermal nevus syndrome (ENS) [2]. Brain hamartomas and Dandy-Walker malformation have been previously reported in separate cases in association with ENS [3]. However, there has been no report in which these lesions were reported in combination with cortical dysplasia. Here we present a unique combination of three different brain lesions in association with ENS, i.e., multiple hamartomas, hypoplasia of the vermis, and dilatation of the 4th ventricle which was consistent with a variant of Dandy-Walker malformation. Informed written consent was obtained from the father of the patient (legal guardian) for this study. No reference to the patient's identity was made at any stage during data analysis or in the report.

## Case presentation

A 25-year-old female with a known diagnosis of epilepsy presented to the emergency department with complaints of fever, anorexia, and uncontrolled convulsions for the last one month. The convulsions  were generalized, tonic-clonic, and were associated with tongue biting, fecal and urinary incontinence as well as  postictal confusion. The seizures lasted for about ten minutes. They were recurrent with episodes occurring on a monthly basis. The fever was of gradual onset, high grade, intermittent in pattern, relieved temporarily by medications and cold sponging. There were no associated rigors or chills. She was reported to be having poor appetite and sleep. She had a history of multiple previous admissions in different hospitals for the management of her seizures. However, no brain imaging had previously been done. Her past medical history was significant for delayed motor, language, and cognitive functions. She was bedridden and needed nursing care since childhood. She had a skin lesion on her face since her birth. There was no significant past surgical history. She was on anti-epileptic medications, but there was no available record.

**Figure 1:**
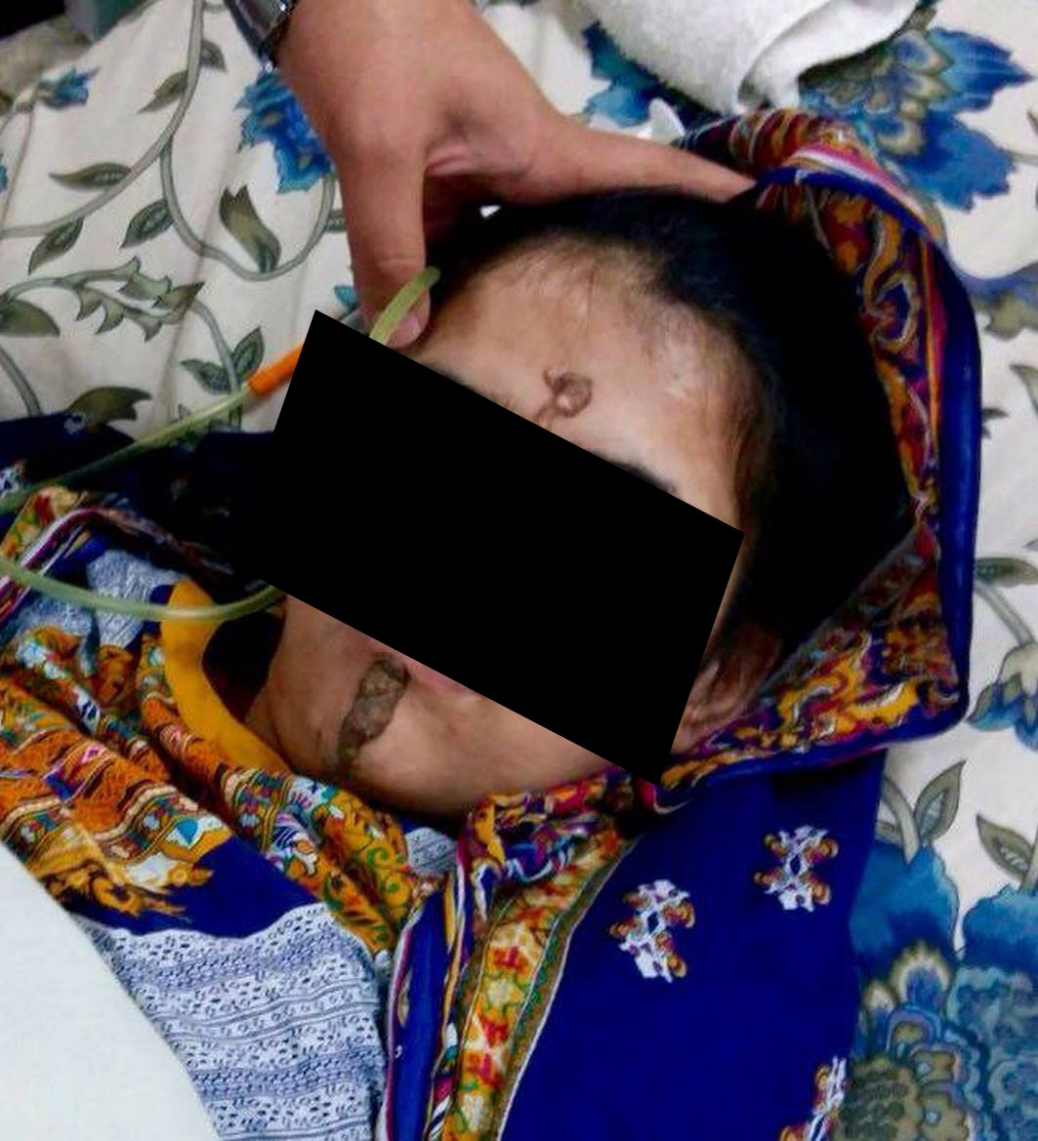
A Dark, Pigmented Epidermal Nevus on the Midline of the Face

On inspection, a young female of average height and build was lying in a drowsy state. She had a dark, pigmented skin lesion on the midline of her face. The lesion was dark brown in color, extending from the submental region to the lower lip and then extending from her nasal bridge to her forehead in the same midline plane (Figure 1).

The lesion was greasy on touch. She was pale and dehydrated. Her temperature was 102^o^ F, pulse was 106 bpm, and her BP was 90/60mm Hg. Her right eye showed many raised dark pigmented lesions on the temporal sides of the optic discs. Both the corneas were hazy in the periphery. She had poor oral hygiene with shallow oral ulcers. Her neurologic examination showed a GCS of 6/15. Her neck was supple with no signs of meningeal irritation. Her pupils were reactive to light. Her plantars were upgoing. Her tone and reflexes were normal. She was aphasic. Her power, sensory system, and cerebellar systems could not be assessed. The rest of her systemic examination was unremarkable. She had a urinary catheter in place.

**Figure 2:**
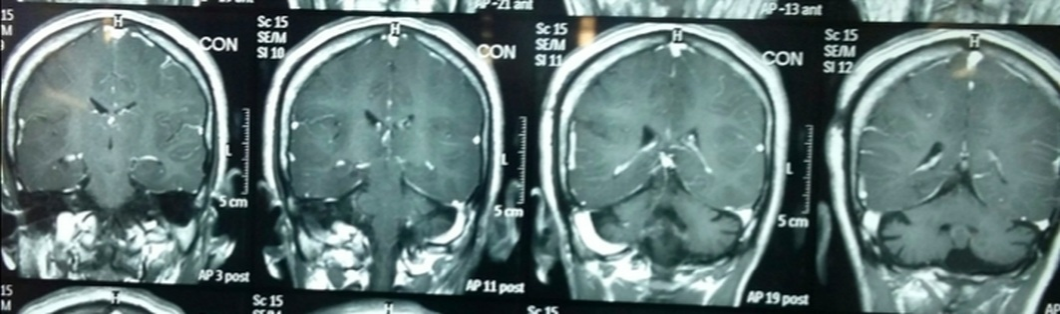
MRI Revealing Cortical Dysplasia and Multiple Hamartomas in the Medial Temporal Lobe, Thalamus, and Periventricular Region

**Figure 3:**
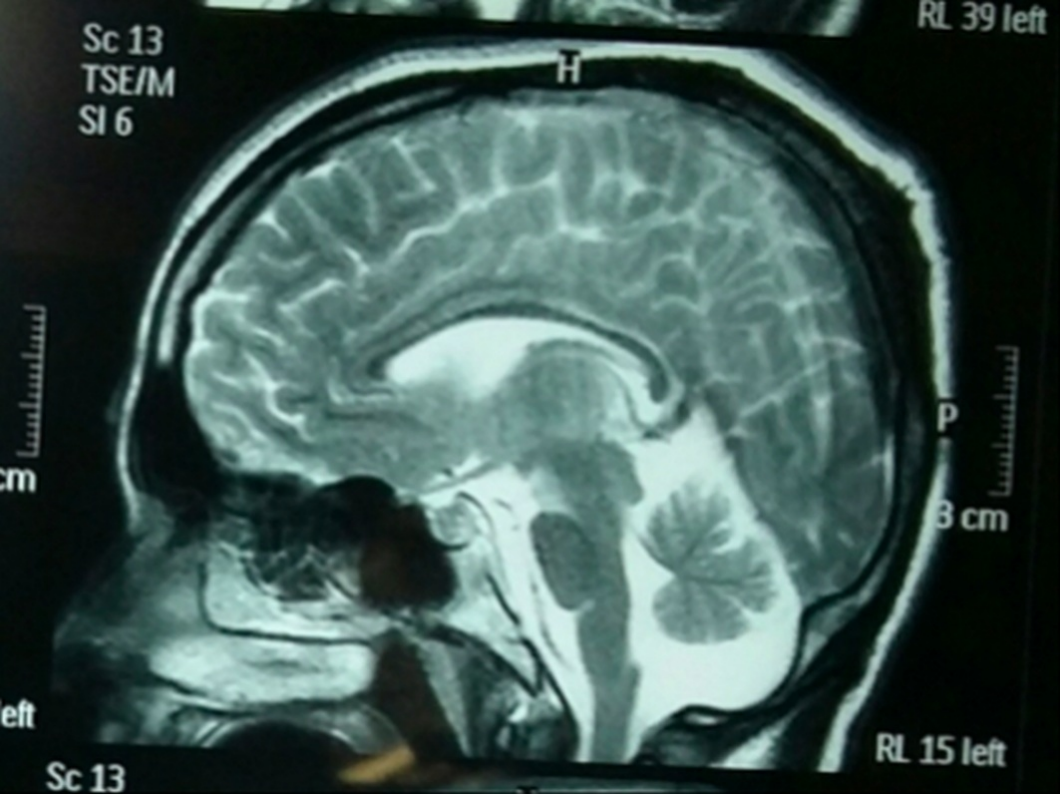
MRI Revealing Cerebellar Atrophy with Dandy-Walker Variant

Laboratory investigation revealed a hemoglobin of 13.4 mg/dL, platelet count of 182,000/mcL, and white blood cell count of 13,000/mcL. The urinalysis showed 8-10 WBCs/HPF and 1-2 RBCs/HPF. Her serum electrolytes, renal function tests, and liver function tests were in the normal range. The urine culture was not reported due to a laboratory error. Her serum calcium was 9.9 mg/dl, and her serum blood glucose level was 120 mg/dL. An ultrasound of the abdomen and pelvis was normal. A skin histopathology was not performed due to the patient's preference. An MRI of the brain with contrast showed congenital cerebral malformations with cortical dysplasia and multiple hamartomas in the medial temporal lobe, thalamus, and periventricular region on right side and cerebellar atrophy with Dandy-Walker variant (Figures 2-3).

On presentation, the differential diagnosis included meningitis, encephalitis, tuberous sclerosis, neurofibromatosis, and Von Hippel-Lindau disease. However, there was no associated neck stiffness, and a lumbar puncture showed no pleocytosis, hyperproteinemia, or hypoglycemia. Furthermore, there were no cafe lait spots, adenoma sebaceum, ash leaf spots, cardiac anomalies, abdominal mass, or eye findings suggestive of the above-mentioned differential diagnosis, and the MRI findings and typical nevus suggested ENS as the most likely diagnosis.

The patient was started on intravenous ceftriaxone and valproic acid for the treatment of the urinary tract infection and seizures, respectively. Her urinary tract infection and seizures resolved by the third day of her hospitalization. She became afebrile, and her GCS score improved to 10/15. The patient was sent home on oral valproic acid, and the family was counseled about hygiene and safety measures to avoid aspiration pneumonia and other complications such as bed sores.

At follow-up after one month, the patient had no change in her cognition, and she still had occasional episodes of tonic-clonic seizures; however, her fever was completely resolved.

## Discussion

ENS is an umbrella term used to represent a group of distinct disorders that are characterized by the presence of one of the different types of epidermal nevi, along with extracutaneous manifestations such as abnormalities of the eyes, nervous, skeletal, and urogenital systems [4]. Epidermal nevi themselves are the hamartomatous overgrowths derived from the various tissues and components of the epidermis. They are classified on the basis of their main component; they may be sebaceous, apocrine, eccrine, follicular, or keratinocytic. They can also be classified as organoid if they have adnexal components (follicular, sebaceous, or apocrine) or non-organoid if they don't have any adnexal components (e.g. keratinocytic). The keratinocytic variant is the most common one [5]. The location of the nevi can provide a clue to the underlying malformation. The presence of epidermal nevi on the head and neck regions can herald abnormalities of the brain, eyes, and cranial bones; whereas those present on the trunk are more likely to be associated with scoliosis, malformation of the hip joint, and deformities of the upper and lower extremities. Our patient had a central nevus on her face which prompted brain imaging thereby identifying brain malformations as the cause of the seizures.

Epidermal nevi occur in approximately 1 to 3 cases per 1,000 live births [6]. ENS has been reported in association with findings such as hemimegaloencephaly, ocular coloboma, ocular dermoid, ocular hemangioma, nephroblastoma, biliary adenoma, hemihypertrophy, cerebral hamartoma and pulmonic stenosis, Dandy-Walker malformation, and vermian hypoplasia amongst several others [7]. Although all three findings have been described separately, this is the first report in which the triad of hamartomas, vermian hypoplasia, and dilation of the 4th ventricle are reported in the same patient.

The presentation and prognosis of ENS vary widely depending on the range and severity of systemic involvement. Primary neurological findings include seizures and mental retardation. Skeletal abnormalities can include spinal scoliosis, malformations of the hip joints, and abnormalities of the upper and lower extremities. Ocular abnormalities may include cataracts, hemangiomas, corneal clouding, or partial absence of tissue of the iris or retina (colobomas) [5]. Endocrine abnormalities such as vitamin D resistant rickets may also occur due to the possible phosphate loss in the urine [8]. Our patient presented with primary neurological findings as one would expect with the location of the nevus on the face.

Management of ENS is individualized and directed toward the symptoms and organ systems involved. It usually requires a multidisciplinary approach provided by a team consisting of a neurologist, dermatologist, ophthalmologist, orthopedic surgeon, physiotherapist, and an internist. Surgical removal of the nevus may be performed, especially if the nevus is sebaceous, as malignant transformation has been reported to occur; however, it is now thought that the risk is overstated. Nevertheless, removal may be performed for cosmetic reasons. Options include full thickness excision of nevi, laser ablation, cryotherapy, or shave ablation depending on the size of nevi [9]. Seizures may be treated with anti-seizure medications. Several cases have been reported where neurosurgery has been required to treat the intractable seizures. Skeletal and ocular malformations may also need surgical intervention. No such efforts were undertaken in our patient based on the patient’s preference. The prognosis of individual ENS varies and depends on the severity of associated underlying deformities.

## Conclusions

ENS describes an association of an epidermal nevus with other neurologic and non-neurologic malformations. Unexplained neurologic symptoms in a patient with a skin lesion should raise suspicion for epidermal nevus syndrome, and imaging studies should be performed to delineate the type and extent of the neurologic malformations so as to guide management.

The location of the nevus can guide the investigations in the most cost-efficient manner. For instance, while dealing with epidermal nevus on the face, associated neurologic and ocular malformations should also be sought after and managed. The neurologic malformations associated with ENS are quite varied and can be very debilitating.
